# Temporal trends and geographical variability of the prevalence and incidence of attention deficit/hyperactivity disorder diagnoses among children in Catalonia, Spain

**DOI:** 10.1038/s41598-020-63342-8

**Published:** 2020-04-14

**Authors:** Laura Pérez-Crespo, Josefa Canals-Sans, Elisabet Suades-González, Mònica Guxens

**Affiliations:** 1 ISGlobal, Barcelona, Spain; 2 Pompeu Fabra University, Barcelona, Spain; 3 Spanish Consortium for Research on Epidemiology and Public Health (CIBERESP), Instituto de Salud Carlos III, Madrid, Spain; 4 Departament of Psychology, CRAMC, Rovira i Virgili University, Tarragona, Spain; 5 Learning Disabilities Unit (UTAE), Neuropediatrics Department, Hospital Sant Joan de Déu, Universitat de Barcelona, Barcelona, Spain; 6 Department of Child and Adolescent Psychiatry/Psychology, Erasmus MC, University Medical Center, Rotterdam, The Netherlands

**Keywords:** ADHD, ADHD, Epidemiology, Epidemiology

## Abstract

Attention deficit/hyperactivity disorder (ADHD) is one of the most common behavioral disorders in childhood. According to a recent systematic review, the worldwide estimate of ADHD prevalence is 7.2% in children. This study aims to assess the prevalence of ADHD diagnoses in 2017 and the incidence of ADHD diagnoses in 2009–2017 in children living in Catalonia, Spain, as well as their temporal and geographical variability, and stratifying by sex and age. We used administrative data for all children aged 4 to 17 years who were insured in the public Catalan Health System in 2009–2017. We identified all ADHD cases diagnosed in 2009–2017 (ICD-9 code 314). We estimated the prevalence of ADHD diagnoses in 2017 and the overall annual incidence of ADHD diagnoses in 2009–2017. We used Poisson regression models to assess temporal trends in the incidence. We estimated a prevalence of ADHD diagnoses of 4.06% (95%CI 4.03, 4.10) in 2017, being 5.81% (95%CI 5.75, 5.87) for boys and 2.20% (95%CI 2.16, 2.24) for girls, the highest prevalence being in 13-to-17-year-olds (7.28% (95%CI 7.20, 7.36)). We did not observe a statistically significant increase of the incidence of ADHD diagnoses during the study period. Geographical differences were found across the healthcare areas in both prevalence and annual incidence and constant during the study period. In conclusion, the prevalence of ADHD diagnoses observed in this study was 4.06%, which was lower than the estimates reported in previous systematic reviews, but in line with the prevalence estimates from other recent European studies. The prevalence was higher in boys than girls, with a sex ratio consistent with previous studies. We did not observe an increase in the temporal trend of incidence of ADHD diagnoses in recent years, but we found geographical differences.

## Introduction

Attention deficit/hyperactivity disorder (ADHD) is one of the most common behavioral disorders in childhood and adolescence^1^. ADHD is commonly characterized by a persistent pattern of inattention, hyperactivity and/or impulsivity. These manifestations can cause a significant impairment in school as well as in the activities of daily life^2^. ADHD appears to have a complex etiology including environmental and genetic risk factors^3^.

In the past decade, several systematic reviews have been conducted to estimate the prevalence of ADHD. According to a recent systematic review conducted with 175 studies across the world, the overall pooled estimate of ADHD prevalence was 7.2% in children^4^. Most of the ADHD prevalence studies were conducted in Europe (54 studies, 31%) and within school populations (130 studies, 74%). The studies included in this review have reported highly variable rates worldwide, ranging from 0.2% to 34.5%. Globally, ADHD prevalence estimates are higher in the Middle East and North America regions and lower in African and Asian countries. Regarding sex differences, most previous studies have shown a higher ADHD prevalence in boys than in girls with an overall sex ratio boy/girl of 2:1 in children^5^. Although ADHD symptoms can be reliably diagnosed since 4 years old^5^, there is no homogeneous evidence among studies about the most prevalent age group of diagnosis. Some studies have reported higher ADHD prevalence in children aged 6–12 years^6–8^. However, some other studies have reported higher ADHD estimates at older age groups^9–11^.

ADHD incidence in children was also assessed in some studies^8,12–15^. Only few studies have reported annual incidence estimates of diagnosed ADHD for an extensive period of time allowing for time trend analysis, but most of them showed an increase in the incidence during the study period^13–15^. Overall, higher incidence was detected in boys and ADHD diagnosis for both boys and girls was greater in children aged 6–11years^14^ and specially among 7–9 years old^13^.

To our knowledge, very few studies with the aim of studying the prevalence of ADHD have been carried out in Spain and there are no studies on the ADHD incidence over an extended period of time^16^. Among the ADHD prevalence studies, only two studies have been conducted in Catalonia over the last years and both among preschoolers^17,18^. However, little is known about the distribution of the prevalence of ADHD diagnoses in other age groups of children in that region and whether the incidence of ADHD diagnoses is still increasing as other studies have reported. Additionally, there is no study conducted in Spain that has evaluated the geographical variability of the prevalence and incidence of ADHD diagnoses. These data are crucial for service planning, resource allocation, training and research priorities, and prevention and treatment programming. Therefore, this study aims to assess the prevalence of ADHD diagnoses in 2017, and its incidence between 2009 and 2017 among the child population of the Catalonia region in Spain, as well as their temporal and geographical variability. Also, this study aims to assess the differences of the prevalence and incidence of ADHD diagnoses by sex and age.

## Results

### Prevalence of ADHD diagnoses in children aged 4 to 17 years in 2017

**Figure 1:**
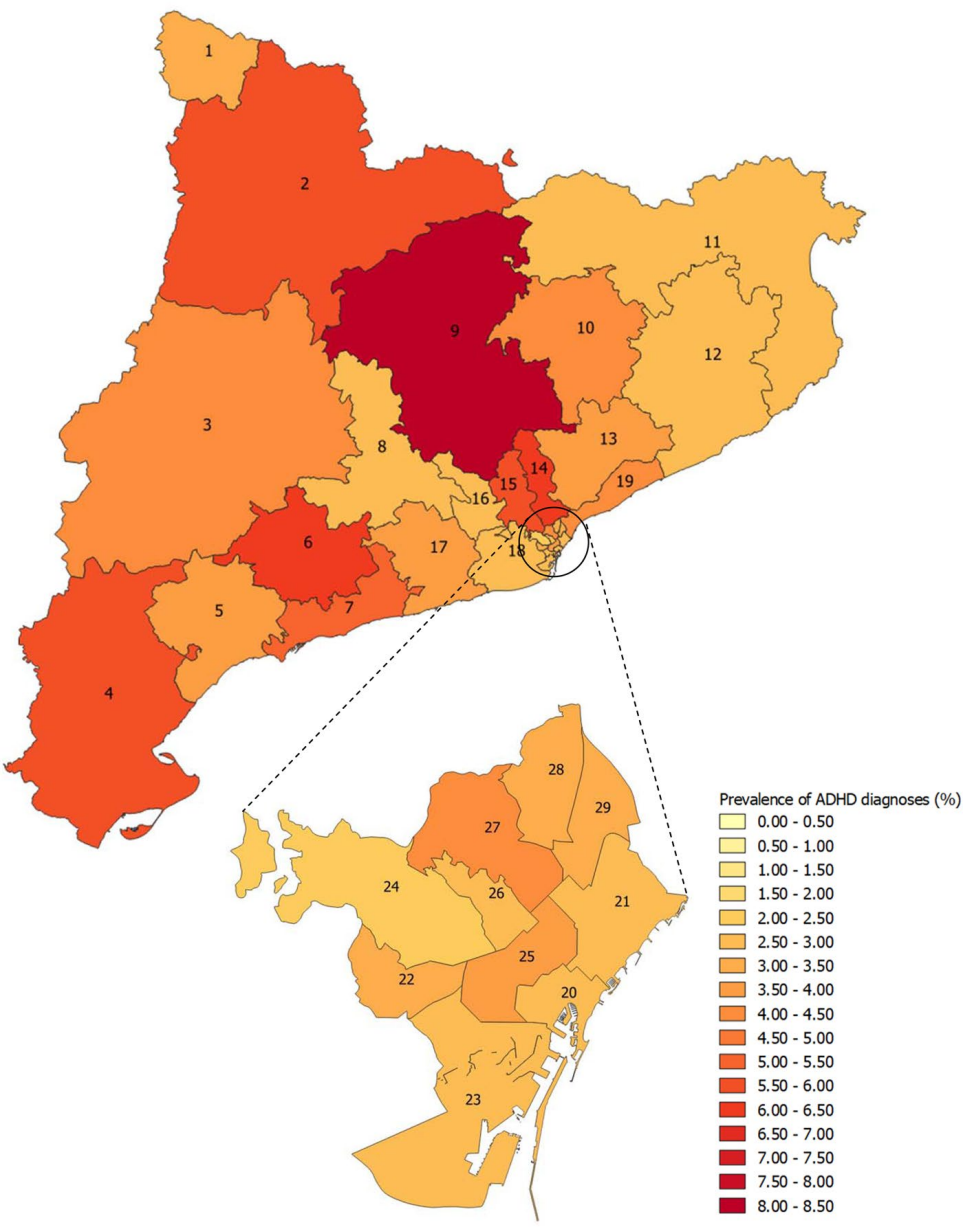
Prevalence proportion of attention deficit/hyperactivity disorder diagnoses (%) among children from 4 to 17 years old in 2017 stratified by the 29 healthcare areas of the Catalonia region, Spain, which are delimited based on geographic, socio-economic, and demographic factors. **1**: Aran; **2**: Alt Pirineu; **3**: Lleida; **4**: Terres de l’Ebre; **5**: Baix Camp- Priorat; **6**: Alt Camp- Conca de Barberà; **7**: Tarragonés- Baix Penedès; **8**: Anoia; **9**: Solsonès- Bages- Berguedà; **10**: Osona; **11**: Girona Nord; **12**: Girona Sud; **13**: Vallès Oriental; **14**: Vallès Occidental Est; **15**: Vallès Occidental Oest; **16**: Baix Llobregat Nord; **17**: Alt Penedès - Garraf; **18**: Baix Llobregat Centre-Litoral i L’Hospitalet de Llobregat; **19**: Barcelonès Nord i Maresme; **20**: Barcelona Ciutat Vella; **21**: Barcelona Sant Martí; **22**: Barcelona les Corts; **23**: Barcelona Sants- Montjuïc; **24**: Barcelona Sarrià- Sant Gervasi; **25**: Barcelona Eixample; **26**: Barcelona Gràcia; **27**: Barcelona Horta- Guinardó; **28**: Barcelona Nou Barris; **29**: Barcelona Sant Andreu. ADHD = Attention deficit/hyperactivity disorder.

In 2017, 1,114,226 children aged 4 to 17 years were covered in the Catalan Health Service, of whom 45,260 had a diagnosis of ADHD. We observed an overall prevalence of ADHD diagnoses of 4.06% (95% CI 4.03, 4.10) in 2017 (Table 1), significantly higher in boys (5.81% (95% CI 5.75, 5.87)) than in girls (2.20% (95% CI 2.16, 2.24)) (p-value < 0.001) with a sex ratio of 2.8-fold (33,349 boys vs. 11,911 girls). We also found significant differences in prevalence of ADHD diagnoses between age groups according to the child’s age in 2017 (p-value < 0.001), with the highest prevalence in the 13 to 17 age group (7.28% (95% CI 7.20, 7.36)) followed by the 7 to 12 years (3.47% (95% CI 3.42, 3.52)), and the 4 to 6 years groups (0.28% (95% CI 0.25, 0.32)). Regarding geographical variability, the prevalence of ADHD diagnoses varied between healthcare areas in 2017 ranging from 2.39% (95% CI 2.19, 2.60) in the Barcelona Sarrià-Sant Gervasi healthcare area to 8.31% (95% CI 8.03, 8.60) in the Solsonès-Bages-Berguedà healthcare area (Fig. 1).

**Table 1:** Prevalence proportion of attention deficit/hyperactivity disorder (ADHD) diagnoses (%) in 2017 and incidence rates of ADHD diagnoses (%) between 2009 and 2017 by sex and age group.

		Total	Sex			Age group^‡^		
			Boys	Girls	Prevalence ratio^†^	4–6 years old	7–12 years old	13–17 years old
**Prevalence**								
**2017**	N	45,260	33,349	11,911	2.8	681	17,350	27,229
	Prevalence Proportion (95% CI)	4.06 (4.03; 4.10)	5.81 (5.75; 5.87)	2.20 (2.16; 2.24)		0.28 (0.25; 0.32)	3.47 (3.42; 3.52)	7.28 (7.20; 7.36)
**Incidence**								
**2009**	N	5,436	4,229	1,207	3.5	590	3,291	1,555
	Incidence Rate (95% CI)	0.51 (0.50; 0.52)	0.77 (0.76; 0.78)	0.23 (0.21; 0.26)		0.23 (0.20; 0.27)	0.73 (0.71; 0.74)	0.43 (0.41; 0.45)
**2010**	N	5,992	4,503	1,489	3.0	650	3,693	1,649
	Incidence Rate (95% CI)	0.56 (0.55; 0.57)	0.82 (0.80; 0.83)	0.29 (0.26; 0.31)		0.25 (0.22; 0.29)	0.81 (0.80; 0.82)	0.46 (0.43; 0.48)
**2011**	N	6,051	4,505	1,546	2.9	582	3,800	1,669
	Incidence Rate (95% CI)	0.56 (0.54; 0.57)	0.81 (0.79; 0.82)	0.29 (0.27; 0.32)		0.22 (0.19; 0.26)	0.81 (0.80; 0.83)	0.46 (0.44; 0.49)
**2012**	N	6,828	4,957	1,871	2.7	610	4,192	2,026
	Incidence Rate (95% CI)	0.63 (0.62; 0.64)	0.88 (0.87; 0.89)	0.35 (0.33; 0.38)		0.23 (0.20; 0.27)	0.88 (0.87; 0.89)	0.57 (0.55; 0.60)
**2013**	N	6,917	5,039	1,878	2.7	448	4,485	1,984
	Incidence Rate (95% CI)	0.63 (0.62; 0.64)	0.89 (0.88; 0.90)	0.35 (0.33; 0.38)		0.17 (0.14; 0.20)	0.94 (0.93; 0.95)	0.56 (0.54; 0.58)
**2014**	N	6,218	4,464	1,754	2.6	437	3,972	1,809
	Incidence Rate (95% CI)	0.56 (0.55; 0.58)	0.78 (0.77; 0.80)	0.33 (0.31; 0.35)		0.17 (0.13; 0.20)	0.82 (0.81; 0.83)	0.50 (0.48; 0.53)
**2015**	N	5,798	4,147	1,651	2.5	414	3,678	1,706
	Incidence Rate (95% CI)	0.53 (0.51; 0.54)	0.73 (0.72; 0.74)	0.31 (0.29; 0.33)		0.16 (0.13; 0.20)	0.76 (0.74; 0.77)	0.48 (0.45; 0.50)
**2016**	N	5,414	3,874	1,540	2.5	428	3,297	1,689
	Incidence Rate (95% CI)	0.49 (0.47; 0.50)	0.68 (0.66; 0.69)	0.29 (0.26; 0.31)		0.17 (0.14; 0.21)	0.66 (0.64; 0.68)	0.46 (0.44; 0.49)
**2017**	N	6,441	4,670	1,771	2.6	513	4,005	1,923
	Incidence Rate (95% CI)	0.58 (0.57; 0.59)	0.81 (0.80; 0.83)	0.33 (0.31; 0.35)		0.21 (0.18; 0.25)	0.80 (0.79; 0.81)	0.51 (0.49; 0.54)

ADHD: attention deficit/hyperactivity disorder; CI: confidence interval; N: number of children with ADHD.

^†^Boys vs. girls.

^‡^Age groups based on child’s age in 2017 for ADHD prevalence and age groups at diagnosis for ADHD incidence.

### Incidence of ADHD diagnoses in children aged 4 to 17 years from 2009 to 2017

**Figure 2:**
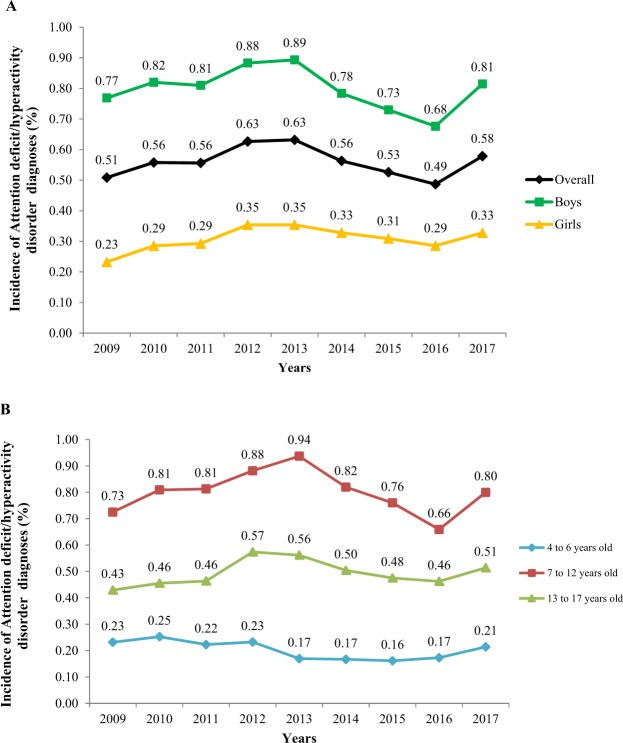
Incidence rate of attention deficit/hyperactivity disorder diagnoses (%) among children from 4 to 17 years old between 2009 and 2017 in the Catalonia region, Spain. (**A**) Overall attention deficit/hyperactivity disorder incidence rate (%) and stratified by sex (**B**) Attention deficit/hyperactivity disorder incidence rate (%) stratified by age group at diagnosis.

**Figure 3:**
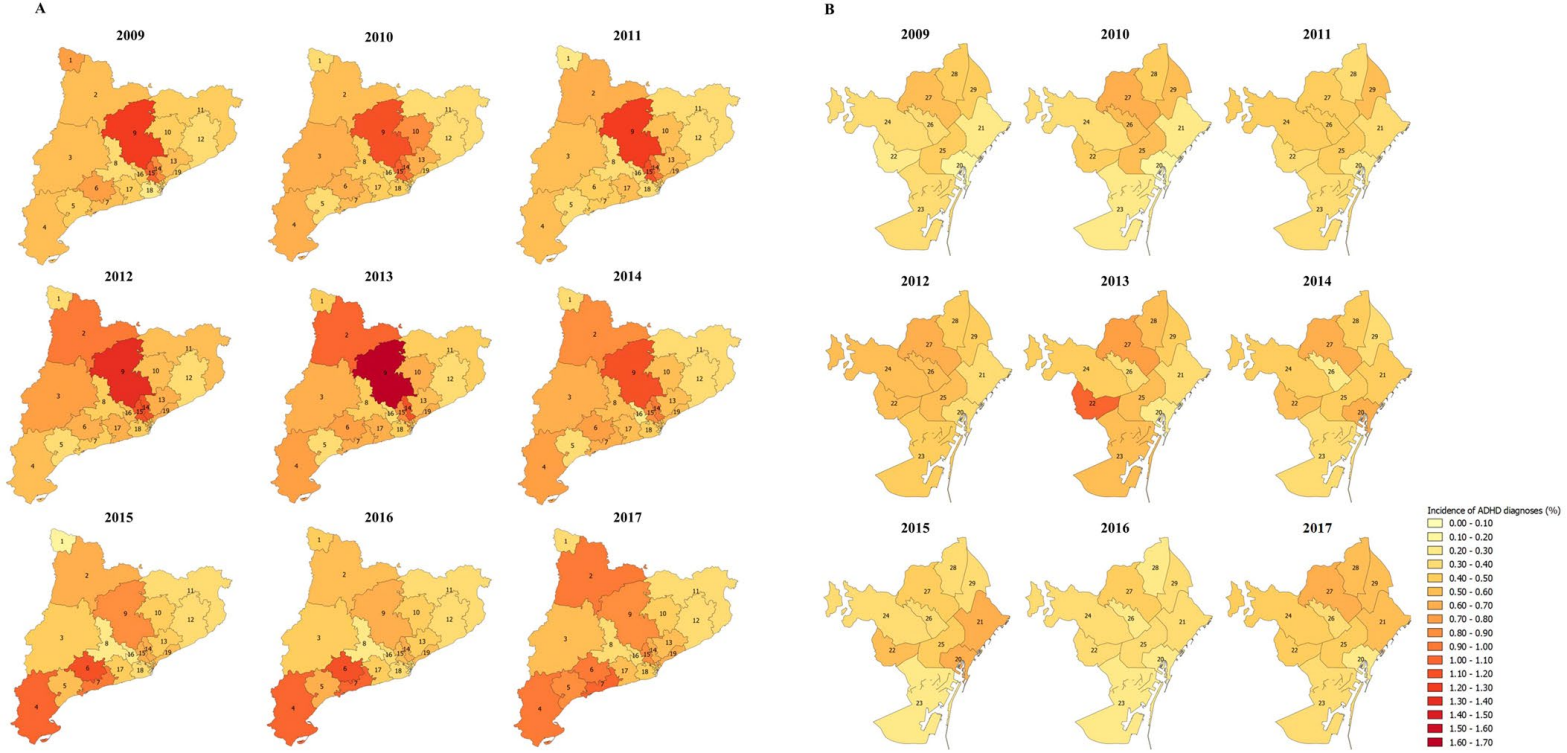
Incidence rate of attention deficit/hyperactivity disorder diagnoses (%) among children from 4 to 17 years old between 2009 and 2017 stratified by the 29 healthcare areas of the Catalonia region, Spain which are delimited based on geographic, socio-economic, and demographic factors. **(A)** Healthcare areas of Catalonia (excluding Barcelona city) **(B)** Healthcare areas of Barcelona city. **1**: Aran; **2**: Alt Pirineu; **3**: Lleida; **4**: Terres de l’Ebre; **5**: Baix Camp- Priorat; **6**: Alt Camp- Conca de Barberà; **7**: Tarragonés- Baix Penedès; **8**: Anoia; **9**: Solsonès- Bages- Berguedà; **10**: Osona; **11**: Girona Nord; **12**: Girona Sud; **13**: Vallès Oriental; **14**: Vallès Occidental Est; **15**: Vallès Occidental Oest; **16**: Baix Llobregat Nord; **17**: Alt Penedès - Garraf; **18**: Baix Llobregat Centre-Litoral i L’Hospitalet de Llobregat; **19**: Barcelonès Nord i Maresme; **20**: Barcelona Ciutat Vella; **21**: Barcelona Sant Martí; **22**: Barcelona les Corts; **23**: Barcelona Sants- Montjuïc; **24**: Barcelona Sarrià- Sant Gervasi; **25**: Barcelona Eixample; **26**: Barcelona Gràcia; **27**: Barcelona Horta- Guinardó; **28**: Barcelona Nou Barris; **29**: Barcelona Sant Andreu. ADHD = Attention deficit/hyperactivity disorder.

During the period between 2009 and 2017, we did not observe a statistically significant increase in the incidence of ADHD diagnoses when we performed Poisson regression models (p-value = 0.111). The incidence of new ADHD cases followed an oscillating nonlinear pattern from 0.51% (95% CI 0.50, 0.52) in 2009 to 0.58% (95% CI 0.57, 0.59) in 2017 (Table 1 and Fig. 2A). However, we observed a slight increase in the incidence of ADHD diagnoses between 2011 (0.56% (95% CI 0.54, 0.57) and 2012 (0.63% (95% CI 0.62, 0.64) but a decrease from 0.63% (95% CI 0.62, 0.64) in 2013 to 0.49% (95% CI 0.47, 0.50) in 2016. In addition, we found the greatest increase in the incidence of ADHD diagnoses in the last year of the study period, between 2016 and 2017. After stratifying by sex, the incidence of ADHD diagnoses was higher in boys compared to girls over all the study period (Table 1 and Fig. 2A) although we could not observe a statistically significant increase of incidence of ADHD diagnoses for any sex. Regarding the age groups at the time of diagnosis, we observed a higher number of new diagnoses of ADHD in children aged 7 to 12 years over all the study period, mainly between the years 2009 and 2013 (Table 1 and Fig. 2B). No statistically significant differences were observed in the temporal trends of incidence when we stratified by age groups. The mean age of ADHD diagnosis was estimated to be 10.4 years, and the median age was 10 years. When studying the incidence of ADHD diagnoses by healthcare areas, we noted geographical differences between healthcare areas for each year of the study period separately. However, when we assessed the temporal trend of incidence from 2009 to 2017, we found that overall incidence of ADHD diagnoses was almost constant within healthcare areas with a slight significant decrease in all of them, except Terres de l’Ebre and Alt Pirineu healthcare areas (Fig. 3 and Supplemental Material Table S1).

## Discussion

This study assessed the prevalence of ADHD diagnoses in 2017 and the incidence of ADHD diagnoses between 2009 and 2017, as well as their temporal and geographical variability and sex and age differences, among children aged 4 to 17 years in the Catalonia region in Spain. The prevalence of ADHD diagnoses in 2017 was estimated at 4.06%, with a sex ratio boy/girl of 2.8:1, and with the highest prevalence in the 13 to 17 age group. We did not observe an increase in the incidence of ADHD diagnoses between 2009 and 2017 although we found a higher number of new cases diagnosed with ADHD among boys and children aged 7 to 12 years. Regarding the geographical analyses, we found differences between healthcare areas in the prevalence of ADHD diagnoses in 2017 and in the annual incidence but the incidence of ADHD diagnoses was almost constant in most healthcare areas during the study period from 2009 to 2017.

Epidemiological studies on ADHD prevalence during childhood and adolescence have reported highly variable estimates worldwide ranging from 0.2% to 34.5%. In the present study, the prevalence of ADHD diagnoses was estimated at 4.06%, indicating a lower estimate than previously reported worldwide values of 5.29%^1^ and 7.20%^4^ in some systematic reviews. However, our findings are in line with the prevalence estimates from other European studies conducted in recent years^11,19–21^, which showed lower prevalence estimates than North American studies^1,4^. The prevalence of ADHD diagnoses found in our study was also lower than the overall ADHD pooled-prevalence of 6.8% reported in a systematic review with studies conducted in Spain^16^. The inconsistency in the prevalence estimates among this systematic review and our study could be explained by some of the following reasons: i) our study included general population of children between 4 and 17 years old while most studies included in the systematic review (78.6%) were conducted using school population with children between 6 and 12 years old, ii) methodology used was different; our study is based on registry data with ADHD diagnoses confirmed by specialized clinicians instead of ADHD confirmation from symptoms referred by parents and teachers as in most of the studies included in the systematic review (64.3%), and iii) our study used ICD-9 code based on DSM-V criteria while most of the studies included in the systematic review using diagnostic criteria applied the DSM-III-R and DSM-IV (71.5%). Overall, the reasons of the variability in the estimates of the ADHD prevalence between and within countries remain poorly understood but age, gender, study design, study period, diagnostic criteria, population assessed, the informant-patient reporting symptoms, sociodemographic factors, and health care access are factors that could have an influence^11^. The different methodologies used in the studies make the comparison of ADHD prevalence estimates very challenging.

With regard to the overall incidence of ADHD diagnoses, incidence estimates have not been on a continuous upwards over the study period from 2009 to 2017. It is unclear whether the increase observed between 2016 and 2017 is just a random variation of the incidence or will be maintained in the following years. Epidemiological studies on the ADHD incidence are published less frequently than those of prevalence, but most of them showed an increasing temporal trend during their respective study periods^8,12,13,22^. However, it is difficult to compare estimates of ADHD incidence from different studies because they may not have been conducted homogeneously: differences in the time periods, geographical locations, and methodological issues. In order to explain the overall changes in the temporal trends of ADHD incidence, some researchers have hypothesized that they might be due to i) an overdiagnosis of ADHD^23^, ii) clinicians that do not follow strictly the diagnostic manuals^24^, iii) changes in ADHD diagnosis criteria that occurred over the study period, iv) an increased awareness in the last years among the parents, teachers, psychiatrists, and pediatricians about ADHD.

ADHD seems to be a predominantly male disorder with a reported sex ratio of approximately 2:1^5^ in children. In our study, we estimated a sex ratio boy/girl of 2.8:1 in the prevalence of ADHD diagnoses in 2017, which is near to the sex ratio reported in the DSM-V. Despite the heterogeneity of the sex ratio reported among different studies, our results are also similar to two studies conducted in 2016 with similar age group populations, in which they found a sex ratio boy/girl of 3.1:1^11^ and 2.3:1^25^, respectively. The organizational effects of sexual hormones such as testosterone during the intrauterine period may influence the development of ADHD predominantly in boys, maybe by acting on the dopaminergic neural system in the prefrontal cortex and striatum influencing the clinical manifestations and severity of ADHD^26^. Emerging evidence however indicates that the stress hormones and sex chromosome genes (i.e X-and Y-linked genes) are also biological mechanisms contributing to sex differences in ADHD^27^. Moreover, girls may be underidentified and underdiagnosed mostly due to differences in the expression of the ADHD symptoms. Girls tend to present more inattentive symptoms and fewer hyperactive/impulsive symptoms as well as fewer disruptive behaviors when compared with boys with ADHD.

The noticeable beginning of ADHD symptoms is often early in childhood, being the behavioral and hyperactivity symptoms those that might already be detected after 4 years old. However, before the age of 4, ADHD symptoms are challenging to diagnose because children have more difficulties to self-regulate their behavior and it is complicated to distinguish ADHD symptoms from normative behaviors. In our study, the highest prevalence of ADHD diagnoses in 2017 was observed in the group of children aged 13 to 17 years with a prevalence of 7.28%. Some studies have also shown higher ADHD prevalence at older age groups. Xu *et al*. found that ADHD prevalence in 2015–2016 in US children and adolescents was higher among adolescents aged 12 to 17 years than children aged 4 to 11 years (13.5% *vs*. 7.7%, respectively)^9^. A study conducted among children and adolescents aged 3 to 17 years diagnosed with ADHD in Germany found that prevalence estimates increased from 1.5% during preschool to 5.3% during elementary school and further increased to 7.1% in secondary school, being the ADHD prevalence higher at older ages^10^. However, our findings differ from some studies that found the highest ADHD prevalence among children aged 6 to 12 years^6,7^. Additionally, we found a lower prevalence of ADHD diagnoses in age group of 4 to 6 years old compared to other studies that reported estimates of 2% to 5%^17,18^. The lower prevalence estimates found in our study in the youngest age group might be explained by reticence among clinicians to label children with ADHD at very young ages or by lack of enough training for health professionals to improve the early diagnosis. Our findings of the incidence of ADHD diagnoses by age groups are consistent with some previous studies carried out in United Kingdom, Canada, Denmark, and Taiwan^8,12–14^. As expected, in our study there was a greater diagnosis of new cases of ADHD over all the study period among children aged 7 to 12 years, which corresponds to the elementary school period where ADHD symptoms tend to be notice and more detectable^28^. These results might be explained by the transition from preschool to elementary schools which involves new demands on child’s organizational skills and reveals difficulties with controlling attention that might have been hidden previously. However, it would be recommended to put more efforts into an early diagnosis and intervention that could significantly reduce the lifelong costs of care for ADHD person. The average estimated total cost of ADHD per year per child was 5732.64€ among Spanish population including medical and non-medical costs^29^.

In the geographical analysis, we found similar results to the study of the prevalence and incidence of ASD diagnoses in children in Catalonia, with a greater incidence of ADHD diagnoses in the central and southern regions^30^. The results of the prevalence of ADHD diagnoses are also in agreement with the results of the geographical variability of the population between 6 to 17 years old that takes medication for ADHD^31^. However, these results should be interpreted with caution since other factors may be associated and affect the geographical variability of ADHD, such as socio-economic factors that might influence the awareness of the disease and therefore the consultation with the specialist, the likelihood of the use of private health versus public health services of each healthcare area, or the different diagnostic and treatment procedures of the medical teams across healthcare areas. Unfortunately, we did not have information of these potential factors that might have influenced the geographical variability of the prevalence and incidence of ADHD and could not investigate the reasons of this variability. Future research to understand the reasons of this variability is warranted in order to develop and implement specific measures aimed at reduce the variability and to improve the diagnostic and treatment procedures in all the region.

The main strengths of our study are the design, which is based on administrative data, and the large sample size included. It is one of the few studies carried out in Southern Europe based on register data. Our study also included data from a relatively long period of time allowing a thorough temporal trend analysis. Finally, we studied the differences in prevalence and incidence of ADHD diagnoses by sex, age group, and across healthcare areas.

Our study has also some limitations. The main limitation is that only ADHD cases from the public healthcare centers that are under the Catalan Health Department were considered. We were unable to access data of those children diagnosed in two other type of centers: i) the private healthcare centers and ii) the Child Development and Early Care Centers that are under the Catalan Welfare Deparment. However, in Catalonia the “Healthy Childhood” program is implemented and visits to the pediatrician are scheduled regularly during the whole childhood^32^. Most of the children covered in the Catalan Health Service follow this program even if they benefit from double health coverage. Therefore, we expect that most ADHD cases have at least one contact with the public healthcare centers in relation to their ADHD diagnosis or for other health issues and that their ADHD diagnosis would be registered. Furthermore, our study is based on register data which only capture individuals who accessed the healthcare services. As a result, participants with the disorder who are not in contact with these services are yet unidentified and not included as cases, leading to a possible underestimation of the prevalence and incidence estimations. In addition, since we are using administrative data we cannot confirm that the ADHD diagnosis assessment has been made in a standard way for all ADHD cases and it could be variation in ADHD diagnostic practices. Although all cases have been identified with ICD-9 code, diagnostic practices might have differed between different facilities or even between healthcare professionals. This potentially contributed to the variation between healthcare areas. It is also possible that in those healthcare areas with higher socioeconomic status, private health services are used more frequently and this could represent an underestimation of the ADHD cases registered. However, as far we known, there are no specific features that may influence the ADHD diagnosis in Catalonia. It has been reported that the percentage of mental health disorders (including ADHD) in Catalonia does not differ from the average of Spain. Other disadvantage is linked to the already defined covering population, time periods, and variables, so it is not possible to include other relevant research questions to the study such as in survey data based studies. Also, data on variables of lesser importance in administrative terms may be of lower quality. Administrative data often lacks any documentation and information about the quality of the data. To our knowledge, no efforts are made by the data provider to perform a case validation of the ADHD cases in the register we used in our study, which precludes us from knowing whether we have an under- or overestimation of the ADHD diagnosis. Further studies focusing on case validation as well as on evaluating the completeness of the data are warranted. However, while we recognize that there are limitations in using a register methodology, other study designs have also important limitations that must be recognized including a low and selected participation of families in the study that would lead to bias estimations or studies relying solely on parental reports may be prone to recall bias^6^. Thus, a methodology using administrative data is less costly and more time-efficient and may provide very large and representative samples of individuals for longtime periods which are not achievable financially or logistically through any survey method. The administrative datasets allow good opportunities for obtaining regional statistics and longitudinal series. Finally, another limitation is that we could not stratify the analyses by the three different subtypes of ADHD recognized, the predominantly inattentive, the predominantly hyperactive/impulsive, and the combined with characteristics of the first two subtypes, since they are not considered in the ICD-9 classification.

## Conclusion

We found a prevalence of ADHD diagnoses of 4.06% in 2017. This prevalence was higher in boys than girls with a sex ratio of 2.8:1, and was highest in the 13 to 17 years age group. We did not observe an increase in the incidence of ADHD diagnoses trends from 2009 to 2017. We observed geographical differences in the prevalence of ADHD diagnoses in 2017 and in the annual ADHD incidence between healthcare areas. ADHD incidence was almost constant within healthcare areas during the study period. This study provides new evidence of the prevalence and incidence of ADHD diagnoses in children in the South of Europe producing essential information for planning and evaluating the needs of the healthcare services as well as the educational services in the Catalonia region.

## Methods

### Population and study design

We used administrative data for children aged 4 to 17 years covered under the Catalan Public Health Service between 2009 and 2017. In this cohort, we identified children diagnosed with ADHD at public healthcare centers between 2009 and 2017, and we defined population-based denominators based on the total population of children in 2017, and the total population of children at risk for each year between 2009 and 2017, as detailed in the following sections. Spain’s National Health System provides universal access to health services for all native and foreign-born children. Therefore, the access to public healthcare centers is available to all children regardless of socioeconomic/financial status or racial/ethnic identification^33^. Catalonia is an autonomous community on the northeastern of Spain. In 2018, the official population of Catalonia was 7,543,825 inhabitants, accounting for about 16.2% of the Spanish population. Catalonia consists of four provinces, Barcelona, Girona, Lleida, and Tarragona, which in turn are divided into 29 healthcare areas which are delimited based on geographic, socio-economic, and demographic factors.

### ADHD cases

In Catalonia, all children diagnosed with ADHD in a public healthcare center are registered by the Catalan Health Service in the Minimum Basic Data Set (MBDS) register. This population register collects information on the patients’ activity and health morbidity, and is populated using information provided by 4 different types of public healthcare centers: mental health centers, mental health outpatient clinics, primary care centers, and hospital discharges. These different centers started to register cases of ADHD at different years. The primary care centers started in 1990 but had good quality and reliable data since 2006, the hospital discharges started in 2005, and the mental health centers and mental health outpatient clinics started in 2008. In 2008, the first year that all centers had available data registered, we observed an expected artifact of high number of ADHD diagnoses registered. Thus we started this study in 2009 when the number of ADHD diagnoses became more stable. If a child was registered with an ADHD diagnosis before 2009, it was excluded from the dataset. The diagnostic procedure of ADHD in any of these centers is carried out following the criteria of the Diagnostic and Statistical Manual of mental disorders 4^th^ edition Text revision (DSM-IV-TR)^34^ and the Diagnostic and Statistical Manual of mental disorders 5^th^ edition (DSM-V)^5^. However, when these diagnoses have to be registered in the MBDS register, they are coded according to the International Classification of Diseases 9^th^ edition (ICD-9)^35^.

Therefore, in this study ADHD cases were defined as those children aged 4 to 17 years with a diagnosis of ADHD between 2009 and 2017 based on the ICD-9 code 314 (hyperkinetic syndrome of childhood). ADHD cases might be duplicated when they are registered in different public healthcare centers or by different health professionals (which happened in 24% of the cases). For our study, we selected the first entry in the register as the first ADHD diagnosis as children have a unique identifier in all centers. We considered the subsequent registered diagnoses of the same child as the same case registered multiple times instead of different ADHD diagnoses. We excluded ADHD cases registered before the age of 4 because ADHD diagnosis before this age might not be reliable^5^.

For each child, we collected information about sex, healthcare area where ADHD was diagnosed, and age at two times: age at diagnosis and age in 2017. Age was stratified in three categories: 4–6 years, 7–12 years, and 13–17 years.

### Population-based denominators

We used the total population of children in 2017 and the total population of children at risk for each year between 2009 and 2017, as registered by the Catalan Health Service through the Central Register of Insured Persons. The total population of children was defined as the number of children aged 4 to 17 years in 2017, and the total population of children at risk as the number of children aged 4 to 17 years who were ADHD-free but still at risk of developing ADHD for each year between 2009 and 2017. For both population-based denominators, we used the population on 1^st^ of January of each year. Both population-based denominators were also stratified by sex, age group, and healthcare areas, using the same category groups as those for the ADHD cases.

### Statistical analyses

We calculated the prevalence of ADHD diagnoses (95% confidence interval (CI)) in 2017 by dividing the number of children aged 4 to 17 years with an ADHD diagnosis that year (numerator) by the total number of children in the same age range in the population in the studied year (denominator). The prevalence in 2017 was stratified by sex, age group (age in 2017), and healthcare area. The differences in prevalence between sexes and age groups were estimated using the Chi-squared test. We plotted the distribution of the prevalence of ADHD diagnoses by healthcare areas in a map. We categorized the distribution of the prevalence of ADHD diagnoses in equal intervals per each 0.50% increase.

We calculated the annual incidence of ADHD diagnoses (95% CI) between 2009 and 2017 by dividing the number of newly diagnosed cases of ADHD among children aged 4 to 17 years (numerator) by the total population at-risk of children aged 4 to 17 years for each year (denominator). Annual incidence between 2009 and 2017 was stratified by sex, age group (at diagnosis), and healthcare area. As there is independence between the occurrences of ADHD events, i.e. one child does not predispose to the appearance of more cases of children diagnosed with ADHD, we used Poisson regression models to assess the temporal trend of the overall incidence between 2009 and 2017, and that for each sex and age group (at diagnosis). Finally, we also plotted in maps the distribution of incidence of ADHD diagnoses for each year and for each healthcare area. We categorized the distribution of the incidence of ADHD diagnoses into deciles.

We used STATA (version 14.2; Stata Corporation, College Station, Texas, USA) and OpenEpi (version 3.01; Dean AG, Sullivan KM, Soe MM) for the statistical analyses. We used QGIS (version 3.0.2 Girona; QGIS Development Team) for creating the maps of the prevalence and incidence by healthcare areas.

### Ethics approval

This study was reviewed and approved by the Clinical Research Ethics Committee of the Parc de Salut Mar that waived the need to obtain informed consent as this study did not involve contact with study subjects, authors only had access to anonymized data through registries. Adequate measures to ensure personal data protection and confidentiality were taken according to the Regulation (EU) 2016/679 on the protection of natural persons with regard to the processing of personal data and on the free movement of such data, and the Spanish Law of Personal Data Protection and Digital Rights Guarantee (3/2018, of 5th December). National regulations on personal data protection were implemented to guarantee the highest standards in personal data management.

## Date availability

The dataset analyzed during the present study is available from the corresponding author on reasonable request.

## Supplementary information

Supplementary Material.
