# Supplementary material for: Temporal trends and geographical variability of the prevalence and incidence of attention deficit/hyperactivity disorder diagnoses among children in Catalonia, Spain
Source: Sci Rep. 2020 Apr 14;10:6397. doi: 10.1038/s41598-020-63342-8 (PMC7156473; doi:10.1038/s41598-020-63342-8)
Supplement: Supplementary file 1 — Supplementary Material. [file 41598_2020_63342_MOESM1_ESM.pdf]

Supplementary Material

Temporal trends and geographical variability of the prevalence and incidence of attention deficit/hyperactivity disorder diagnoses among children in Catalonia, Spain

Laura Pérez-Crespo, Josefa Canals Sans, Elisabet Suades-Gonzalez, Mònica Guxens

Table S1. Prevalence proportion of attention deficit/hyperactivity disorder (ADHD) diagnoses (%) in 2017 and incidence rates of ADHD diagnoses (%) between 2009 and 2017 among children from 4 to 17 years old by healthcare areas of the Catalonia region, Spain……………………………………2

Table S2. Number of children aged 4-17 in Catalonia for each year between 2009 and 2017, overall and stratified by sex and age groups……………………………………………………………..7

Table S1. Prevalence proportion of attention deficit/hyperactivity disorder (ADHD) diagnoses (%) in 2017 and incidence rates of ADHD diagnoses (%) between 2009 and 2017 among children between 4 to 17 years old by healthcare areas of the Catalonia region

	Prevalence	Incidence								
**Healthcare area**	**2017**	**2009**	**2010**	**2011**	**2012**	**2013**	**2014**	**2015**	**2016**	**2017**
**Lleida**										
N	2378	263	306	303	375	346	340	252	242	294
Rate	4.50	0.53	0.61	0.59	0.73	0.67	0.65	0.48	0.46	0.56
(95% CI)	(4.33; 4.68)	(0.47; 0.59)	(0.56; 0.66)	(0.54; 0.65)	(0.68; 0.77)	(0.62; 0.72)	(0.60; 0.70)	(0.42; 0.54)	(0.40; 0.52)	(0.50; 0.61)
**Alt Camp-Conca de Barberà**										
N	554	64	56	36	56	71	73	101	109	89
Rate	6.00	0.72	0.63	0.40	0.62	0.78	0.80	1.10	1.18	0.96
(95% CI)	(5.54; 6.51)	(0.61; 0.83)	(0.50; 0.76)	(0.24; 0.56)	(0.49; 0.75)	(0.69; 0.88)	(0.71; 0.89)	(0.91; 1.34)	(0.98; 1.42)	(0.93; 1.00)
**Tarragonès - Baix Penedès**										
N	2879	260	279	250	230	359	320	496	554	594
Rate	5.32	0.54	0.56	0.49	0.44	0.69	0.60	0.93	1.03	1.10
(95% CI)	(5.14; 5.52)	(0.48; 0.60)	(0.51; 0.62)	(0.43; 0.55)	(0.38; 0.51)	(0.64; 0.73)	(0.55; 0.66)	(0.91; 0.95)	(0.95; 1.12)	(1.01; 1.19)
**Baix Camp-Priorat**										
N	1236	147	112	97	120	128	121	187	215	266
Rate	3.86	0.48	0.36	0.31	0.38	0.40	0.37	0.58	0.67	0.83
(95% CI)	(3.66; 4.08)	(0.40; 0.56)	(0.27; 0.45)	(0.22; 0.40)	(0.29; 0.46)	(0.31; 0.48)	(0.29; 0.46)	(0.51; 0.65)	(0.61; 0.73)	(0.79; 0.88)
**Terres de l' Ebre**										
N	1497	151	169	153	144	193	207	264	264	242
Rate	5.99	0.59	0.66	0.59	0.55	0.74	0.80	1.03	1.04	0.97
(95% CI)	(5.70; 6.29)	(0.51; 0.66)	(0.59; 0.73)	(0.51; 0.67)	(0.47; 0.63)	(0.68; 0.80)	(0.74; 0.85)	(0.92; 1.16)	(0.91; 1.17)	(0.95; 0.99)
**Girona Nord**										
N	1510	213	192	198	265	242	204	187	193	190
Rate	2.90	0.42	0.39	0.39	0.52	0.47	0.40	0.36	0.37	0.36
(95% CI)	(2.76; 3.05)	(0.36; 0.49)	(0.32; 0.45)	(0.32; 0.46)	(0.46; 0.58)	(0.41; 0.54)	(0.33; 0.46)	(0.29; 0.43)	(0.30; 0.44)	(0.30; 0.43)
**Girona Sud**										
N	2089	234	256	300	284	286	277	314	253	302
Rate	2.60	0.31	0.34	0.39	0.36	0.37	0.35	0.40	0.32	0.38
(95% CI)	(2.49; 2.71)	(0.25; 0.37)	(0.28; 0.39)	(0.33; 0.44)	(0.31; 0.42)	(0.31; 0.42)	(0.29; 0.41)	(0.34; 0.45)	(0.26; 0.37)	(0.32; 0.43)

Table S1 (continuation). Table S1. Prevalence proportion of attention deficit/hyperactivity disorder (ADHD) diagnoses (%) in 2017 and incidence rates of ADHD diagnoses (%) between 2009 and 2017 among children between 4 to 17 years old by healthcare areas of the Catalonia region

	Prevalence	Incidence								
**Healthcare area**	**2017**	**2009**	**2010**	**2011**	**2012**	**2013**	**2014**	**2015**	**2016**	**2017**
**Anoia**										
N	497	52	83	67	71	79	93	51	50	65
Rate	2.73	0.30	0.48	0.38	0.40	0.44	0.52	0.28	0.27	0.36
(95% CI)	(2.51; 2.98)	(0.18; 0.43)	(0.37; 0.59)	(0.27; 0.50)	(0.29; 0.52)	(0.33; 0.55)	(0.42; 0.62)	(0.16; 0.41)	(0.15; 0.40)	(0.24; 0.47)
**Osona**										
N	1009	113	197	138	126	149	140	107	106	118
Rate	4.06	0.48	0.84	0.57	0.52	0.61	0.57	0.44	0.43	0.47
(95% CI)	(3.82; 4.31)	(0..39; 0.58)	(0.79; 0.89)	(0.49; 0.66)	(0.44; 0.61)	(0.53; 0.69)	(0.49; 0.65)	(0.34; 0.53)	(0.33; 0.52)	(0.38; 0.56)
**Solsonès- Bages- Berguedà**										
N	3028	412	376	421	459	581	430	324	250	294
Rate	8.31	1.23	1.11	1.21	1.30	1.64	1.20	0.90	0.69	0.81
(95% CI)	(8.03; 8.60)	(1.12; 1.35)	(1.00; 1.22)	(1.10; 1.33)	(1.19; 1.43)	(1.51; 1.77)	(1.09; 1.32)	(0.86; 0.93)	(0.63; 0.75)	(0.76; 0.85)
**Aran**										
N	38	10	5	3	4	6	4	2	6	3
Rate	3.03	0.72	0.36	0.22	0.30	0.49	0.32	0.16	0.47	0.32
(95% CI)	(2.22; 4.14)	(0.44; 1.00)	(-0.06; 0.78)	(-0.25; 0.68)	(-0.15; 0.75)	(0.09; 0.89)	(-0.14; 0.77)	(-0.35; 0.66)	(0.07; 0.87)	(-0.14; 0.78)
**Alt Pirineu**										
N	476	45	46	58	82	89	70	55	46	75
Rate	5.97	0.54	0.55	0.68	0.98	1.07	0.85	0.67	0.57	0.94
(95% CI)	(5.47; 6.51)	(0.39; 0.68)	(0.40; 0.69)	(0.56; 0.80)	(0.94; 1.01)	(0.87; 1.32)	(0.76; 0.93)	(0.55; 0.80)	(0.42; 0.71)	(0.89; 0.99)
**Baix Llobregat Nord**										
N	756	106	94	104	143	100	75	77	91	97
Rate	2.68	0.43	0.37	0.40	0.54	0.37	0.27	0.28	0.32	0.34
(95% CI)	(2.50; 2.87)	(0.33; 0.52)	(0.27; 0.47)	(0.31; 0.49)	(0.46; 0.62)	(0.28; 0.47)	(0.17; 0.37)	(0.18; 0.38)	(0.23; 0.42)	(0.25; 0.44)
**Vallès Oriental**										
N	2866	354	440	363	467	409	387	351	338	407
Rate	3.88	0.51	0.63	0.51	0.66	0.57	0.53	0.48	0.46	0.55
(95% CI)	(3.74; 4.02)	(0.46; 0.56)	(0.58; 0.67)	(0.46; 0.56)	(0.62; 0.70)	(0.52; 0.62)	(0.48; 0.58)	(0.43; 0.53)	(0.41; 0.51)	(0.50; 0.60)

Table S1 (continuation). Table S1. Prevalence proportion of attention deficit/hyperactivity disorder (ADHD) diagnoses (%) in 2017 and incidence rates of ADHD diagnoses (%) between 2009 and 2017 among children between 4 to 17 years old by healthcare areas of the Catalonia region

	Prevalence	Incidence								
**Healthcare area**	**2017**	**2009**	**2010**	**2011**	**2012**	**2013**	**2014**	**2015**	**2016**	**2017**
**Vallès Occidental oest**										
N	4379	696	708	837	786	632	620	501	435	595
Rate	5.91	1.02	1.01 (0.94;	1.18	1.11	0.88	0.85	0.69	0.59	0.80
(95% CI)	(5.74; 6.08)	(0.94; 1.09)	1.09)	(1.10; 1.26)	(1.03; 1.19)	(0.86; 0.91)	(0.82; 0.88)	(0.65; 0.73)	(0.54; 0.64)	(0.77; 0.84)
**Vallès Occidental est**										
N	4225	530	589	572	765	741	621	454	453	446
Rate	6.29	0.86	0.95 (0.93;	0.92	1.19	1.14	0.94	0.69	0.68	0.66
(95% CI)	(6.11; 6.47)	(0.83; 0.89)	0.97)	(0.90; 0.94)	(1.11; 01.28)	(1.06; 1.22)	(0.92; 0.96)	(0.65; 0.73)	(0.67; 0.72)	(0.62; 0.71)
**Barcelona Ciutat Vella**										
N	276	20	22	30	44	28	73	71	27	30
Rate	2.71	0.16	0.18 (0.02;	0.24	0.37	0.25	0.65	0.68	0.26	0.29
(95% CI)	(2.41; 3.04)	(0.00; 0.32)	0.35)	(0.09; 0.39)	(0.23; 0.52)	(0.09; 0.41)	(0.54; 0.76)	(0.57; 0.79)	(0.10; 0.43)	(0.13; 0.46)
**Barcelona Sant Martí**										
N	859	59	80	98	95	112	143	196	110	160
Rate	2.90	0.20	0.28 (0.18;	0.33	0.32	0.38	0.49	0.67	0.37	0.54
(95% CI)	(2.71; 3.09)	(0.10; 0.31)	0.37)	(0.24; 0.43)	(0.23; 0.42)	(0.29; 0.47)	(0.40; 0.57)	(0.60; 0.74)	(0.28; 0.46)	(0.46; 0.62)
**Barcelona les Corts**										
N	305	25	39	29	52	91	48	50	35	41
Rate	3.42	0.26	0.41 (0.26;	0.31	0.56	1.01	0.54	0.56	0.39	0.46
(95% CI)	(3.06; 3.82)	(0.08; 0.43)	0.57)	(0.14; 0.47)	(0.43; 0.70)	(0.82; 1.23)	(0.39; 0.68)	(0.42; 0.70)	(0.23; 0.56)	(0.31; 0.61)
**Barcelona Sants - Montjuïc**										
N	510	81	50	83	97	118	65	58	45	76
Rate	2.56	0.37	0.23 (0.11;	0.38	0.46	0.58	0.32	0.29	0.23	0.38
(95% CI)	(2.35; 2.79)	(0.26; 0.47)	0.35)	(0.28; 0.49)	(0.36; 0.56)	(0.49; 0.66)	(0.21; 0.43)	(0.17; 0.41)	(0.10; 0.35)	(0.27; 0.49)
**Barcelona Sarrià - Sant Gervasi**										
N	507	82	86	92	121	99	87	63	78	87
Rate	2.39	0.37	0.39	0.42	0.57	0.47	0.41	0.30	0.37	0.41
(95% CI)	(2.19; 2.60)	(0.27; 0.48)	(0.29; 0.50)	(0.32; 0.52)	(0.48; 0.66)	(0.38; 0.57)	(0.31; 0.52)	(0.19; 0.41)	(0.26; 0.48)	(0.31; 0.51)

Table S1 (continuation). Table S1. Prevalence proportion of attention deficit/hyperactivity disorder (ADHD) diagnoses (%) in 2017 and incidence rates of ADHD diagnoses (%) between 2009 and 2017 among children between 4 to 17 years old by healthcare areas of the Catalonia region

	Prevalence	Incidence								
**Healthcare area**	**2017**	**2009**	**2010**	**2011**	**2012**	**2013**	**2014**	**2015**	**2016**	**2017**
**Barcelona Eixample**										
N	955	147	165	148	153	157	140	126	103	134
Rate	3.52	0.47	0.54	0.49	0.53	0.56	0.50	0.46	0.38	0.49
(95% CI)	(3.31; 3.75)	(0.39; 0.56)	(0.47; 0.62)	(0.41; 0.57)	(0.45; 0.61)	(0.48; 0.64)	(0.42; 0.58)	(0.37; 0.55)	(0.28; 0.47)	(0.41; 0.58)
**Barcelona Gràcia**										
N	342	50	75	62	66	49	39	41	39	40
Rate	2.57	0.37	0.55	0.45	0.50	0.37	0.30	0.32	0.30	0.30
(95% CI)	(2.31; 2.85)	(0.23; 0.50)	(0.44; 0.67)	(0.33; 0.58)	(0.38; 0.62)	(0.24; 0.51)	(0.15; 0.44)	(0.17; 0.46)	(0.15; 0.44)	(0.16; 0.44)
**Barcelona Horta - Guinardó**										
N	864	106	129	94	130	148	121	89	97	132
Rate	4.39	0.51	0.63	0.46	0.65	0.75	0.61	0.45	0.49	0.67
(95% CI)	(4.11; 4.68)	(0.41; 0.60)	(0.55; 0.71)	(0.36; 0.56)	(0.56; 0.73)	(0.68; 0.82)	(0.52; 0.70)	(0.35; 0.56)	(0.39; 0.59)	(0.59; 0.75)
**Barcelona Nou barris**										
N	693	88	98	79	94	117	98	80	51	116
Rate	3.29	0.42	0.47	0.38	0.45	0.56	0.47	0.39	0.25	0.55
(95% CI)	(3.06; 3.54)	(0.32; 0.52)	(0.37; 0.57)	(0.27; 0.49)	(0.35; 0.55)	(0.47; 0.65)	(0.37; 0.57)	(0.28; 0.49)	(0.13; 0.36)	(0.46; 0.64)
**Barcelona Sant Andreu**										
N	606	88	94	91	74	83	68	66	60	99
Rate	3.28	0.49	0.53	0.51	0.41	0.47	0.38	0.37	0.33	0.54
(95% CI)	(3.04; 3.55)	(0.39; 0.60)	(0.43; 0.63)	(0.41; 0.61)	(0.30; 0.53)	(0.36; 0.57)	(0.27; 0.50)	(0.25; 0.48)	(0.21; 0.45)	(0.44; 0.63)
**Alt Penedès-Garraf**										
N	1524	157	185	198	250	248	223	179	170	215
Rate	3.79	0.43	0.49	0.52	0.65	0.63	0.56	0.45	0.42	0.54
(95% CI)	(3.61; 3.99)	(0.35; 0.50)	(0.42; 0.57)	(0.45; 0.59)	(0.59; 0.71)	(0.57; 0.69)	(0.50; 0.63)	(0.38; 0.52)	(0.35; 0.50)	(0.47; 0.60)

Table S1 (continuation). Table S1. Prevalence proportion of attention deficit/hyperactivity disorder (ADHD) diagnoses (%) in 2017 and incidence rates of ADHD diagnoses (%) between 2009 and 2017 among children between 4 to 17 years old by healthcare areas of the Catalonia region

	**Prevalence**	**Incidence**								
**Healthcare area**	**2017**	**2009**	**2010**	**2011**	**2012**	**2013**	**2014**	**2015**	**2016**	**2017**
**Baix Llobregat Centre - Litoral i l'Hospitalet de Llobregat**										
N	4046	387	570	535	615	620	580	490	446	589
Rate	2.89	0.29	0.42	0.40	0.45	0.45	0.42	0.35	0.32	0.42
(95% CI)	(2.80; 2.98)	(0.24; 0.33)	(0.38; 0.46)	(0.35; 0.44)	(0.41; 0.49)	(0.41; 0.49)	(0.38; 0.46)	(0.31; 0.40)	(0.28; 0.36)	(0.38; 0.46)
**Barcelonès Nord i Maresme**										
N	4356	496	491	612	660	636	551	566	548	645
Rate	4.08	0.49	0.48	0.59	0.63	0.61	0.52	0.54	0.51	0.60
(95% CI)	(3.97; 4.20)	(0.44; 0.53)	(0.44; 0.52)	(0.55; 0.63)	(0.60; 0.67)	(0.57; 0.65)	(0.48; 0.56)	(0.50; 0.58)	(0.47; 0.56)	(0.57; 0.64)

CI: 95% confidence interval; N: number of children with ADHD.

Table S2. Number of children aged 4-17 in Catalonia for each year between 2009 and 2017, overall and stratified by sex and age groups

		Sex		Age group		
	**Total**	**Boys**	**Girls**	**4-6 years**	**7-12 years**	**13-17 years**
**2009**	1,069,698	550,315	519,383	254,642	452,906	362,150
**2010**	1,074,487	552,636	521,851	256,073	456,209	362,205
**2011**	1,087,767	559,733	528,034	260,396	467,302	360,069
**2012**	1,090,096	561,380	528,716	261,943	475,143	353,010
**2013**	1,094,766	564,017	530,749	263,630	478,330	352,806
**2014**	1,104,742	569,520	535,222	261,883	484,467	358,392
**2015**	1,102,520	568,147	534,373	256,197	487,416	358,907
**2016**	1,111,946	572,771	539,175	247,444	499,388	365,114
**2017**	1,114,226	573,671	540,555	239,462	500,637	374,127
